# Toward a proposal for a national strategy for access to outdoor stays in residential care facilities for older adults in Sweden

**DOI:** 10.3389/fpsyg.2026.1651502

**Published:** 2026-07-21

**Authors:** Anna Bengtsson, Helene Landin, Madeleine Liljegren, Göran Lindahl, Susanna Nordin, Helle Wijk

**Affiliations:** 1 Department of People and Society, The Swedish University of Agricultural Sciences, Alnarp, Sweden; 2 Institute of Health and Care Sciences, University of Gothenburg, Gothenburg, Sweden; 3 Department of Architecture and Civil Engineering, Chalmers University of Technology, Gothenburg, Sweden; 4 Centre for a DEMentia-Friendly Society with a Person-Centred Mindset, Gothenburg, Sweden; 5 Center for Healthcare Architecture, Chalmers University of Technology, Gothenburg, Sweden; 6 School of Health and Welfare, Dalarna University, Falun, Sweden; 7 Department of Quality Assurance and Patient Safety, Sahlgrenska University Hospital, Gothenburg, Sweden

**Keywords:** collaborative workshop, long-term care, nursing home, older adults, outdoor environment, outdoor access, residential care facility, strategy proposal

## Abstract

**Background:**

Research consistently demonstrates that access to nature plays a significant role in supporting health and wellbeing, particularly among older adults and individuals experiencing illness. Research reviews show that treatment interventions in outdoor environments have positive effects on older adults’ health, quality of life, and physical capacity, that socialization and cognitive ability are promoted, and that stress and depression can be reduced. Despite this knowledge, there is currently no national strategy for access to outdoor environments and outdoor stays at residential care facilities for older adults in Sweden.

**Purpose:**

The purpose of this paper is to propose a national strategy, along with ideas for implementation, developed through a collaborative workshop process.

**Methods:**

The study comprised three collaborative digital workshops, i.e., participants worked with the researchers leading the process. The participants were purposefully sampled and included researchers and representatives from relevant authorities and organizations, as well as stakeholders, practitioners, and key players in residential care facilities for older adults. Five researchers oversaw the iterative process, which lasted for one and a half years. Workshop 1 established a shared foundation and generated initial inputs, which informed the development of a draft proposal. This draft was refined through feedback and discussions in Workshop 2 and further elaborated in Workshop 3 with a focus on implementation strategies. Thus, the research team synthesized the outcomes from all workshops through an iterative process, resulting in the final version of the national strategy proposal.

**Results:**

The results present visions and proposals structured around four policy areas: 1. Outdoor stays; 2. Outdoor care and rehabilitation; 3. The outdoor environment; 4. Knowledge transfer. The results contain detailed descriptions for each of the policy areas, as well as proposals for how to realize the policy areas in a Swedish context.

**Discussion:**

We do not know of any country that has developed a similar national strategy, and therefore, in the final part of this paper, we discuss the generalizability of the strategy, as well as how it relates to person-centered care.

## 1 Introduction

People worldwide are living longer than ever before in history, which calls for a new public health approach to population aging (World Health Organization [WHO], 2025). Extensive research shows that access to and contact with nature is of great importance for people’s health and wellbeing (Nilsson and Grahn, 2024; White et al., 2019; World Health Organization. Regional Office for Europe, 2016), not least in old age and for those experiencing frailty (Magnussen et al., 2021; Ottosson and Grahn, 2005; White et al., 2019). Despite this knowledge, there is a lack of established strategies to ensure access to and contact with nature for older adults in residential care facilities (RCFs) (Fumagalli et al., 2017; Artmann et al., 2017; Heród et al., 2022). The present study forms part of the Swedish research project OUT-FIT (University of Gothenburg, 2025a), and is one of several studies examining how outdoor environments in residential care facilities (RCFs), as a form of urban nature, can promote health and wellbeing among older adults and care staff in their professional practice. Within the OUT-FIT project, outdoor environments are defined to include physical settings such as balconies, patios, conservatories, and gardens, as well as surrounding areas with natural elements, such as public squares, walkways, parks, and other green spaces. Further, outdoor stays refer to general outdoor exposure, without a predefined therapeutic or rehabilitative purpose. Such exposure may include recreational, restorative, or everyday interactions with outdoor or natural environments and is not necessarily structured, supervised, or goal-directed within a healthcare context. In contrast, outdoor care and rehabilitation refer to organized and purpose-driven care and rehabilitation activities in which outdoor settings are intentionally incorporated to support specific health-related or functional outcomes. These practices are typically characterized by therapeutic planning, professional guidance, and explicit rehabilitative objectives, where the outdoor environment could serve as an integrated component of the rehabilitation process rather than solely as the setting in which activities occur.

As part of the project framework, an international overview has been conducted to gain information about how different countries are addressing the issue of older adults’ access to outdoor environments and outdoor activities in RCFs (University of Gothenburg, 2025b). The results indicate a variation in the level of awareness, initiatives, and approaches in a selection of European countries, which is confirmed in a study by Artmann et al. (2017). The absence of a strategic approach in Sweden has, among other things, the consequence that even though care workers and older adults in RCFs see the value of outdoor environments and wish to use them, access is lacking both environmentally and organizationally (Liljegren, 2025). This suggests that the implementation of nature-based approaches currently depends largely on local priorities, individual engagement, and available resources, resulting in unequal access between facilities and municipalities. The lack of national guidelines, long-term funding structures, and coordinated responsibilities may therefore constitute structural barriers to a more systematic and equitable integration of outdoor environments in RCF care practices. The present paper describes the development of a proposal for a Swedish strategy and gathers ideas for how to realize the proposal. This introduction presents evidence that both motivates the need for such a strategy and constitutes the evidence base for the proposal. This evidence base is general in nature and not specific to the Swedish RCF context.

Firstly, the strategy proposal is based on evidence about health benefits from being in natural environments. According to White et al. (2019), spending at least 2 h a week in a natural environment is associated with self-rated good health and wellbeing for people in general. Particularly large health benefits from exposure to natural environments (e.g. gardens and neighborhood greenness) have been demonstrated for vulnerable groups, such as older adults, persons experiencing stress, the sick, those socioeconomically disadvantaged, or persons going through some form of life crisis (Flowers et al., 2016; Mitchell and Popham, 2008; Ottosson and Grahn, 2005, 2006; Weimann et al., 2015; White et al., 2019). The health effects are evident both through self-assessment and with objective measures (Li et al., 2021; White et al., 2019; Zhang et al., 2017). Connectedness with nature as well as access to green areas seem to be crucial, since both aspects affect the number of outdoor visits per week as well as the number of minutes per week that people spend outside in green areas (Fleury-Bahi et al., 2023).

Secondly, the proposed strategy is informed by evidence linking physical activity to health benefits, as the positive impact of access to nature is partly mediated by increased opportunities for movement in varied outdoor environments (Flowers et al., 2016; Weimann et al., 2017; Cardinali et al., 2024). The importance of physical activity for physical, mental, and cognitive health and wellbeing is well documented, e.g., by Bauman et al. (2016); the International Society for Physical Activity and Health (2017), and Wong et al. (2023). According to the World Health Organization [WHO] (2025), all adults, including older people, should engage in some form of heart-rate-increasing physical activity for 150–300 min per week. The International Association of Gerontology and Geriatrics-Global Aging Research Network (IAGG-GARN) has developed concrete recommendations for RCFs, which include movement breaks two to three times a day and varied exercise training for 30–45 min at least twice a week (de Souto Barreto et al., 2016).

Furthermore, natural environments afford neutral places for social interaction and restoration, both of which are important pathways linking nature contact to human health (Markevych et al., 2017). Research shows that RCF gardens, both in Europe and specifically in Sweden, are primarily used for socialization and relaxation (Artmann et al., 2017; Dahlkvist et al., 2020).

The strategy proposal is also founded on McCormack et al.’s (2021) person-centered practice framework (originally termed person-centered). Today, healthcare facilities internationally are increasingly adopting person-centered care approaches (McCormack et al., 2021; Rosengren et al., 2021) that respond to the individual’s life story, resources, needs, preferences, and values in a holistic way (Ebrahimi et al., 2021; Phelan et al., 2020). For many people, contact with nature is an existential need (Passmore and Howell, 2014), and according to Tornstam (2005), interest in nature even increases with age. Studies by Liljegren et al. (2024a, 2024b) show that both older adults and care workers in RCFs acknowledge the value of using outdoor environments as arenas for person-centered care and rehabilitation, recognizing their positive impact on health and wellbeing. In line with this, several research reviews show that care and rehabilitation interventions in outdoor environments have positive effects on the health, quality of life, and physical capacity of older adults, that socialization and cognitive abilities are promoted, and that stress and depression can decrease (Chu et al., 2019; Heród et al., 2022; Nicholas et al., 2019; Yun et al., 2023). Moreover, studies increasingly demonstrate good results from care and rehabilitation interventions in outdoor environments for older adults living with dementia, such as improved perception of mental health and identity (Blake and Mitchell, 2016) as well as improved mood and social interaction (Evans et al., 2019). To align with the concept of person-centered care (McCormack et al., 2021), just as for interventions indoors, it is important that interventions are selected and led by staff, that they are specifically tailored to each person, and that the outdoor environment has a purposeful design and content.

In addition, contact with nature and outdoor activities have been shown to be significant in workplaces such as hospitals (Cordoza et al., 2018; Iqbal and Abubakar, 2022). Research studies indicate that views of greenery can have a positive impact on work capacity, job satisfaction, and creativity (Lottrup et al., 2013; Plambech and Konijnendijk van den Bosch, 2015), and that opportunities for outdoor activities during breaks contribute to better stress recovery and reduce sick leave among care staff (Cordoza et al., 2018). Nature-based activities in dementia care also have positive effects on staff retention (Evans et al., 2019).

**FIGURE 1:**
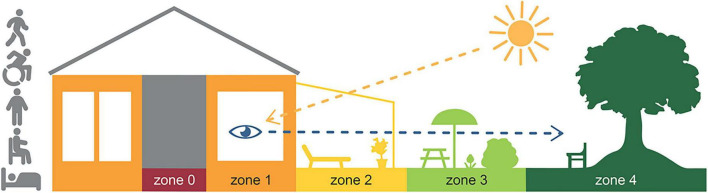
The principal model of four zones of contact with the outdoors. Illustration by Anna Bengtsson.

For the strategy to comprehensively utilize the health-promoting potential of contact with nature, it is also based on the principal model of four zones of contact with the outdoors (Bengtsson, 2015). The model points out four zones in the physical environment in which health-promoting contact with the outdoors can take place (see Figure 1; Bengtsson, 2015; Bengtsson et al., 2025). Access to daylight and views through windows (zone 1) offer potential for health and wellbeing and can be significant for promoting outdoor activities on balconies or patios (zone 2), in the garden (zone 3), or in parks and natural areas in the surroundings (zone 4) (Bengtsson et al., 2025). Zone 0 consists of spaces in the building without contact with the outdoor environment, i.e., windowless spaces. Reviews of the evidence concerning health-promoting contact with the outdoors in each of the four zones are presented in previous publications by Bengtsson (2015) and Bengtsson et al. (2025).

Since posture and mobility affect contact and interaction with the outdoors, the zone model also incorporates a range of physical positions, including lying, sitting, standing, and ambulating independently or with support. Thus, these body positions as well as variations in cognitive function need to be considered. This is particularly relevant in a RCF context, as older adults’ functional capacity and health status vary widely, with many residents experiencing significant physical and cognitive impairments that affect mobility and daily functioning, while others may have less severe limitations that still influence their care needs and opportunities for engagement. Such heterogeneity in functional and cognitive status is well documented among older adults living in care settings, in Sweden and internationally (Socci et al., 2023). Within the Swedish welfare context, RCF constitute a needs-assessed form of municipally provided residential care. These facilities are intended for older adults with extensive care needs and offer 24-h access to care staff (Innes, 2020).

Overall, the research presented above emphasizes the need to establish strategies for access to and contact with nature for older adults in RCFs and demonstrates that a good interplay between indoor and outdoor environments, according to the zones in Figure 1, is significant for older adults’ and care workers’ health and wellbeing in RCFs. As RCFs struggle with limitations in time and personnel resources (American Health Care Association, 2022; Mukamel et al., 2023), it seems crucial to develop effective and health-promoting methods in these settings where the outdoor environment is integrated as a natural part of the daily environment of the older adults as well as in the work environment of the staff. There is therefore reason to conclude that in such improvement efforts, guidelines for physical and social activity can be combined with evidence of the health-promoting effects of the outdoor environment to create added value.

Based on this research, the aim of this study is to develop a proposal for a national strategy for access to outdoor environments and outdoor stays in RCFs for older adults in Sweden and to gather ideas on how the proposal can be implemented. The proposal itself is directed toward politicians and decision-makers and is intended to serve as a foundation for recommendations and guidelines for access to outdoor environments and outdoor activities for older adults living in RCFs in Sweden. The strategy proposal, along with the working process that led to its development and the ideas for implementation, aims to serve as inspiration for countries that lack such strategies.

## 2 Materials and methods

The research approach used to develop the strategy proposal for access to outdoor environments and outdoor stays in RCFs was grounded in an overarching qualitative methodology. It draws on research through designing (Lenzholzer et al., 2013) and participatory action research (PAR) (Baum et al., 2006) to enable an iterative and context-sensitive exploration of how physical environments support health and wellbeing, everyday practices, and care routines. These approaches are inherently compatible with collaborative workshop methods (see Ørngreen and Levinsen, 2017; Li et al., 2020, for descriptions of these methods), which facilitate their participatory and iterative qualities. Further, the collaborative workshop method was chosen to, as described by Ørngreen and Levinsen, 2017, enhance meaning negotiation and to identify factors that are not obvious to either participants or researchers beforehand. Moreover, this method is particularly well suited to studies that aim to foster cooperation and co-creation among multiple stakeholders (Li et al., 2020), as was the case in this study.

Given the ambition to develop a novel strategy at the national level, engaging a diverse range of stakeholders with varying perspectives and geographical conditions, a flexible mode of collaboration was required. An online format enables participants to engage in a shared space and cultivate a community of practice regardless of physical location (Ørngreen and Levinsen, 2017). The process was therefore conducted digitally.

### 2.1 Participants

**FIGURE 2:**
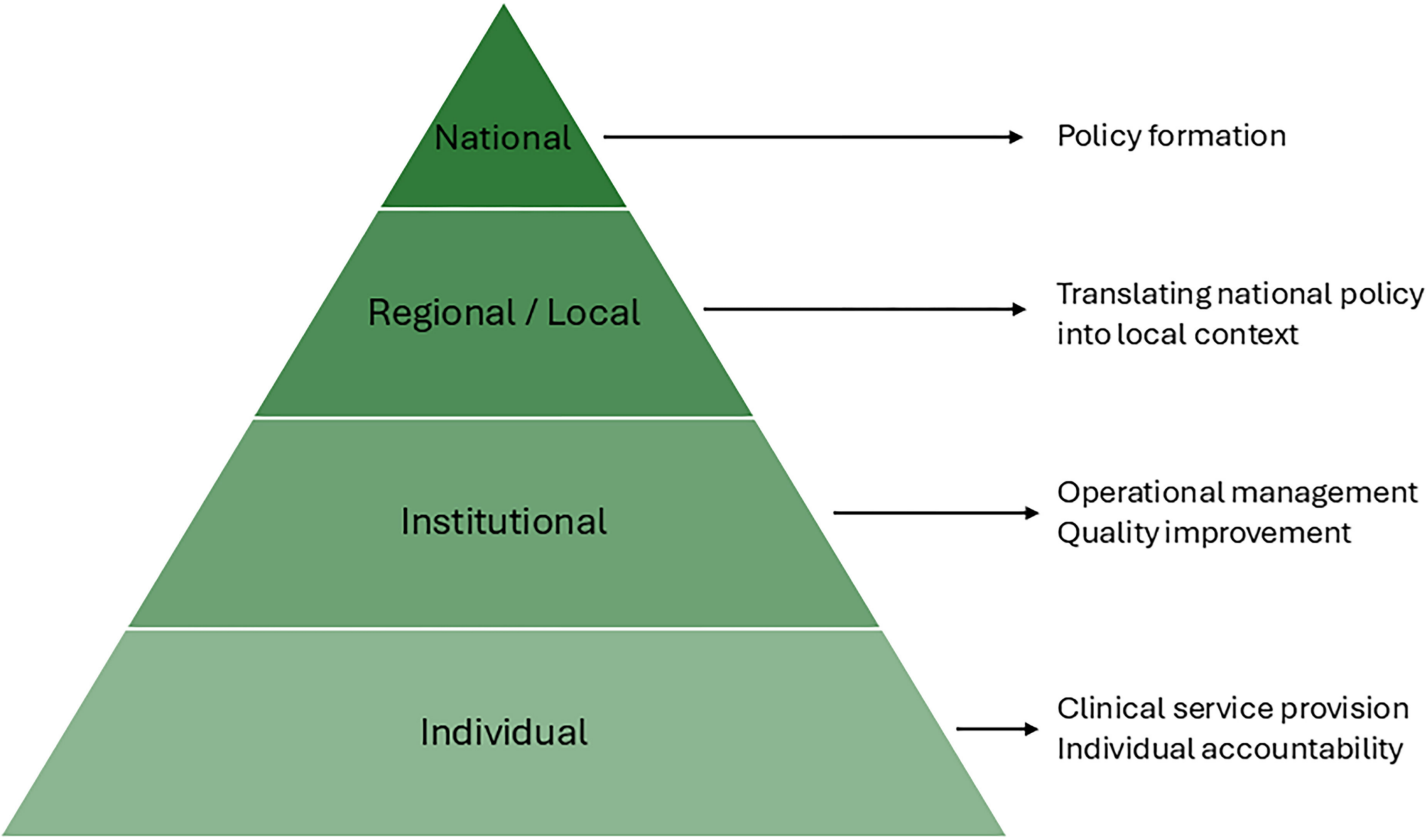
The multi-level model for building capacity for a national quality strategy. Adaptation of the model of Molloy et al. (2016).

The participants were purposefully sampled to include a wide range of stakeholders relevant to developing a national strategy in the domain of RCFs for older adults. The multi-level model for building capacity for a national quality strategy (Kislov et al., 2014; Molloy et al., 2016) was used to identify participants at all crucial levels, from the individual level to the national level (see Figure 2). More than a hundred people were invited to each workshop several months in advance, and reminders were sent out two weeks, and again two or three days, before the workshops took place.

Representatives from each level described in Figure 2 participated in each workshop (see Table 1). In addition, many participants represented more than one, or even several levels. For example, most participants had either worked in RCFs and/or had family or friends living in RCFs. However, in Table 1, each participant is only included once.

**TABLE 1:** Participants in the three workshops, according to the multi-level model for building capacity for a national quality strategy, developed by Kislov et al. (2014) and Molloy et al. (2016).

Level:	National level	Regional/local level	Institutional level	Individual level
**Level description from original sources:**	Persons working with policy formulation, resourcing, infrastructure, and accountability to the public	Persons working with the translation of national policy into the local context, macro-management, and monitoring	Persons working with governance, operational management, and quality improvement	The level of encounter between patients and health professionals
**Participants representing each level at each workshop in the present study:**	Politicians, representatives from national authorities, researchers, and academics	Employees at regional or municipal strategic units	Employees with strategic work tasks related to one or more RCF	Care workers with work tasks at one or more RCF, family and friends of older adults in RCFs
**Number of participants representing each level at workshop 1:**	23	7	6	6
**Number of participants representing each level at workshop 2:**	11	5	3	5
**Number of participants representing each level at workshop 3:**	8	3	2	5

The purposive sampling approach ensured the inclusion of stakeholders with relevant expertise and experience, contributing to a diverse and contextually grounded material. Furthermore, the iterative and participatory nature of the workshop process, aligned with the research through designing approach (Lenzholzer et al., 2013), strengthened the study by enabling continuous dialogue, reflection, and the integration of multiple perspectives throughout the research process.

### 2.2 Collaborative workshop process

In a collaborative workshop process, researchers and participants work together, with the researchers leading the process (Ørngreen and Levinsen, 2017). In this study, a research team of five researchers oversaw the collaborative workshop process, which lasted for one and a half years, from September 2023 to February 2025, and consisted of three workshops. The interdisciplinary research team comprised experts from the disciplines of healthcare science, environmental psychology, architecture, and landscape architecture.

**FIGURE 3:**
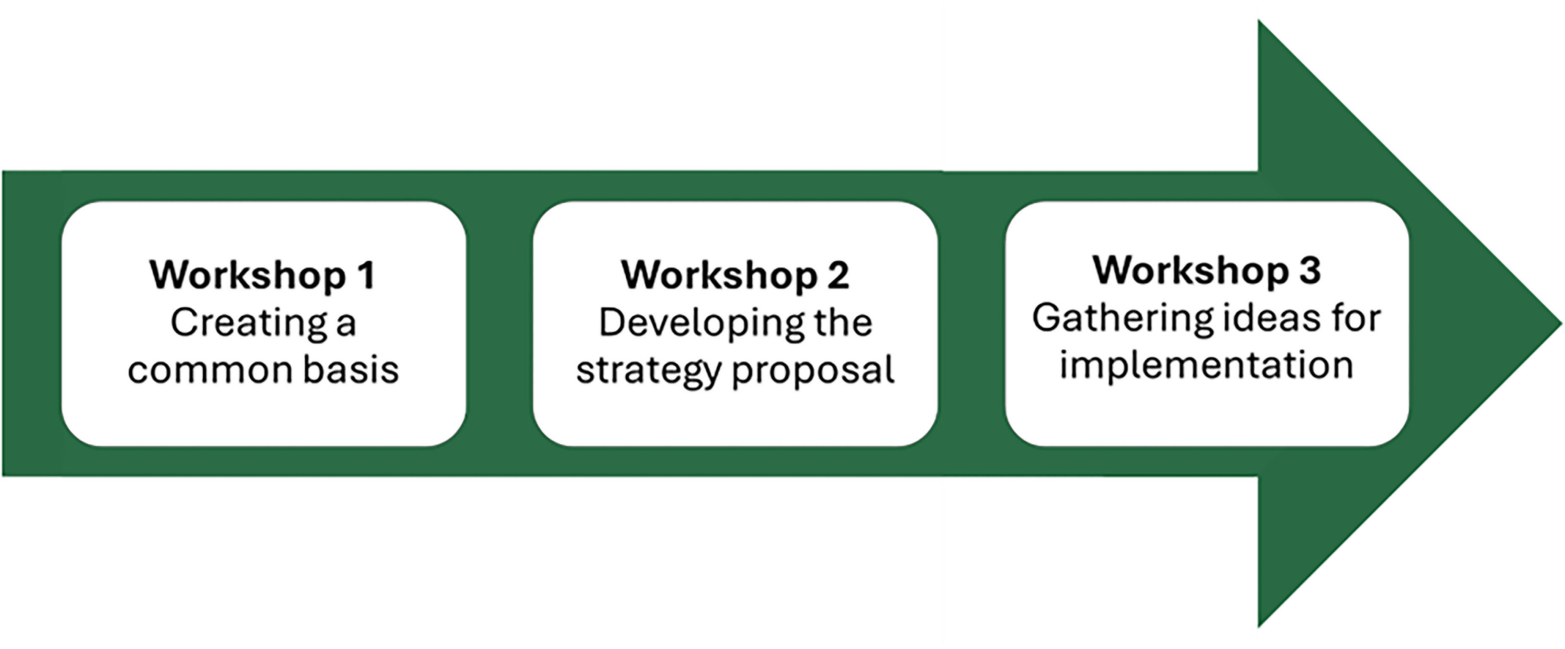
The main steps of the workshop process. Illustration by Anna Bengtsson.

The combination of perspectives from different fields aimed to broaden the analytical lens and to reduce the risk of single-discipline bias in both the facilitation and interpretation of the work. Two researchers (i.e., authors 1 and 2) planned, moderated, and documented the three workshops, and regular check-ins took place with the entire research team. The workshop process thus used a conceptual format with pre-designed phases in a series of workshops, aiming to generate progression over time (Öberg and Hernvall, 2016; Ørngreen and Levinsen, 2017). The involvement of researchers with diverse disciplinary backgrounds further supported critical reflection throughout the process, including in the design, moderation, and documentation of the workshops, as well as in the subsequent analysis. The process was set up following the main steps illustrated in Figure 3 and as described in the following sub-sections.

#### 2.2.1 Workshop 1: Creating a common basis for the strategy proposal

The intention of the first workshop was to create a common basis for the strategy work. Therefore, participants in the workshop representing academia (i.e., from environmental psychology, architecture, landscape architecture and healthcare research), authorities (The National Board of Health and Welfare and the National Board of Housing and Building), lived experience (the Dementia Association), practice (healthcare and rehabilitation professionals), law and politics (a legal expert and a member of the Swedish Parliament) gave presentations relating to the overall theme, outdoor environments and outdoor stays in RCFs for older adults and to the following five sub-themes:

- Sub-theme 1: Outdoor stays in RCFs for older adults.
- Sub-theme 2: Outdoor care and rehabilitation in RCFs for older adults.
- Sub-theme 3: The outdoor environment in RCFs for older adults.
- Sub-theme 4: Knowledge transfer to care workers and managers in RCFs for older adults.
- Sub-theme 5: Law and politics: The legal conditions and political processes.

During the workshop, the participants were asked to use the chat function for reflections on the overall theme of the workshop and on the five sub-themes that the interdisciplinary research group had developed for the workshop. The reflection prompts were:

- Overall theme: Why and in what ways are outdoor environments and outdoor stays in RCFs important?
- Sub-theme 1: Reflect on how to define outdoor stays, and on the possibility of arranging outdoor stays in RCFs for older adults.
- Sub-theme 2: Reflect on how to define outdoor rehabilitation, and on the possibility of outdoor rehabilitation in RCFs for older adults.
- Sub-theme 3: Reflect on and give suggestions concerning outdoor environments and access to outdoor environments in RCFs for older adults.
- Sub-theme 4: Reflect on and give suggestions concerning knowledge transfer to care workers and managers in RCFs.
- Sub-theme 5: Reflect on how to ensure access to outdoor environments, outdoor stays, and outdoor rehabilitation in RCFs.

The workshop outputs were documented through saved presentations, chat notes and written reflections by the research team, compiled immediately after the session. In addition, supplementary reflections were collected via email between workshops and included in the dataset. These materials formed the basis for a qualitative, descriptive thematic synthesis conducted by the researchers.

The material was first organized according to the predefined sub-themes (1–4) and subsequently discussed iteratively within the interdisciplinary research team. Through repeated comparative discussions across the documentation sources, preliminary interpretations were developed and refined into an initial draft proposal for a national strategy.

Consensus was achieved through iterative group discussion and triangulation of interpretations within the research team rather than through formal voting procedures. Divergent interpretations were discussed until convergence was reached, and in cases where full agreement was not initially achieved, formulations were revised and re-evaluated collectively until consensus was obtained.

No formal qualitative coding software was used; however, the analysis followed a structured and systematic thematic approach, moving from documentation to interpretation and synthesis. This process also led to a refinement of the thematic structure, including the integration of the original overarching theme and sub-theme 5 into the other sub-themes, resulting in four consolidated policy areas.

The initial proposal included visions and suggestions for each policy area and was sent out to all participants for review before workshop 2.

#### 2.2.2 Workshop 2: Developing the strategy proposal

The intention of the second workshop was to further develop the strategy proposal. Therefore, each of the four policy areas was presented and reflected upon by one expert (see Table 2).

**TABLE 2:** Experts reflecting on each policy area in workshop 2.

Policy area	Outdoor stays in RCFs for older adults	Outdoor care and rehabilitation in RCFs for older adults	The outdoor environment in RCFs for older adults	Knowledge transfer to care workers and managers in RCFs for older adults
Expert reflecting	A development secretary from a municipality in which a strategy for outdoor stays in RCFs has been developed	A dementia nurse from a municipality that works strategically to individually tailor walks for older adults with dementia	A planning leader in a municipality in which guidelines for governing key figures for garden areas in RCFs have been developed	A lecturer in healthcare research at a Swedish University

After each of the four presentations, participants were divided into five groups, with five people in each group. Each group was assigned a breakout room and an online whiteboard. The vision and proposals for each policy area, as well as prepared templates to work in, were pre-posted on the whiteboards, and all groups were asked to comment on the content, wording, and relevance of all four policy areas in four different sessions.

Based on the documentation from the second workshop, the research group iteratively refined the draft proposal through systematic comparison of participant input and the existing thematic structure, leading to further consolidation and revision of the proposed strategy areas. Throughout this process, interpretations and revisions were discussed iteratively within the research team until consensus was achieved. The final strategy proposal is presented in the results sections 3.1–3.4 and was sent out to all participants before workshop 3.

#### 2.2.3 Workshop 3: Gathering ideas for implementation of the strategy proposal

The third workshop aimed at gathering ideas for the implementation of the strategy proposal. Therefore, relevant authorities were identified for each policy area, and representatives were asked to prepare presentations in which they reflected on the proposal from the perspective of the authority they represented, and considered what steps and measures would be needed for its realization (see Table 3). After that, several presentations followed, with examples of strategic work in relation to outdoor stays, activities, and environments in RCFs.

**TABLE 3:** Overview of policy areas and authorities, with representatives presenting and reflecting on the proposal from the perspective of the authority they represented.

Policy areas	Outdoor stays in RCFs for older adults	Outdoor care and rehabilitation in RCFs for older adults	The outdoor environment in RCFs for older adults	Knowledge transfer to care workers and managers in RCFs for older adults
Authority	The Institute for Human Rights	The National Board of Health and Welfare	The National Board of Housing, Building, and Planning	The National Board of Health and Welfare

The last part of the workshop was devoted to group work. The moderating researchers divided the participants into four groups, with one group leader in each group. Each group was assigned to one of the policy areas 1–4. The groups were then given a template (see Table 4) with the vision and proposal for their policy area and were asked to identify what was needed for implementation to take place and how it could be achieved.

**TABLE 4:** Template for group work to compile ideas for implementation of the strategy proposal.

National strategy for access to outdoor environments and outdoor stays for older adults in residential care facilities in Sweden Vision: Policy area:		1 Date:
2 Name of the person taking notes during the group discussion: **3** (Save a copy of the document and share during presentations in the main meeting room)		
HOW can the proposal be implemented?		
WHAT actions need to be done?	WHEN will this take place?	WHO is expected to take the initiative? (Including: What role can I play?)

The groups began working on their respective policy areas during the group work session and then presented their ideas and received feedback from the larger workshop group. After the workshop, each group had 2 months to complete the compilation of ideas in a document. All documents were sent to the research team before the deadline.

A qualitative content analysis was conducted in line with Schreier (2012) since this method of analysis offers a structured yet flexible way to analyze qualitative data tailored to the research goals. The method combines concept-driven and data-driven analysis through the eight steps described in Table 5.

**TABLE 5:** Description of eight steps to analyze data from the group work, in line with Schreier (2012).

1. Define research question	2. Select material	3. Build coding frame	4. Define coding units	5. Test the coding frame	6. Evaluate coding frame	7. Conduct main analysis	8. Interpret and report results
Two research questions were defined: What is needed for implementation to take place? How can this be done? (According to the groups)	The texts produced by the groups at the end of the assignment period were selected	A concept-driven and data-driven coding frame was developed	The coding was tailored to the data and the goal of the study	The coding frame was tried out on parts of the data and then revised	The reliability and validity of the coding frame were carefully considered	The final version of the coding frame was applied to the entire data set	The findings were presented in the results and reflected upon during the discussion

Authors 1 and 2 conducted the initial analysis, after which the results were reconsolidated and further refined through an iterative process of discussion and critical reflection within the interdisciplinary research team, ensuring both that participant input and diverse disciplinary perspectives were incorporated. The results in section 3.5 thus present themes of statements expressing what needs to be done for the strategy to be realized, and (if relevant) who should do it.

The final results reflect an iterative and collaborative process in which research-based knowledge and stakeholder contributions were systematically integrated through repeated cycles of discussion, comparison, and refinement. The outputs should be understood as collectively generated results from a structured co-creation process, rather than as empirically tested or validated findings. Accordingly, they represent a synthesized proposal informed by both scientific evidence and stakeholder perspectives.

### 2.3 Ethics

Ethical approval was not required for this study, as it did not involve the collection of personal or sensitive data. Participation in the online workshops was voluntary, and no personal data are reported in the results.

## 3 Results

The results are presented in two parts. First, the visions and proposals for a national strategy based on a structure of four policy areas are presented. Then, ideas for how to implement the strategy proposal are presented in four themes.

### 3.1 Policy area 1: Outdoor stays at residential care facilities for older adults

*Vision:* Spending time outdoors is a natural part of everyday life in RCFs. Since many older adults cannot enter or exit buildings on their own or initiate outdoor activities themselves, providing daily outdoor time for residents who wish to participate is included in the care work. The care workers and older adults are well acquainted with the outdoor environment of their residence and the activities that are available there.

*Proposal:* The government should initiate support for the implementation of outdoor stay strategies across all RCFs in Sweden. The strategy should include the following principles regarding outdoor stays:

- All older adults living in RCFs should be able to use each of the four zones as often as they wish, as independently as possible.
- Individual needs and preferences regarding outdoor activities should be essential considerations in the implementation plan.
- Knowledge about the importance of outdoor environments, outdoor stays, and outdoor care and rehabilitation should be linked to the RCF’s core values and included in ongoing work (team meetings, workshops, etc.), so that all care workers are involved and can contribute with their personal knowledge and experiences.
- Each RCF should establish a routine for scheduling and planning outdoor activities throughout the year.
- Everyone living at an RCF should be offered outdoor time for at least 2 h per week (either continuous or on different occasions), preferably in combination with meals, social activities, or physical activities.

### 3.2 Policy area 2: Outdoor care and rehabilitation at residential care facilities for older adults

*Vision:* Outdoor-based care and rehabilitation permeate the work and are carried out by all care workers. It is just as natural to utilize the outdoor environment as the indoor environment for activities and interventions in RCFs.

*Proposal:* The government should initiate support for the implementation of outdoor stay strategies across all RCFs in Sweden. The strategy should encompass the following principles regarding outdoor care and rehabilitation:

- Routines for outdoor care and rehabilitation should be developed and implemented.
- Care and rehabilitation interventions should be integrated into the outdoor environment, as well as indoor settings, when preferred by the older adult, throughout the year.
- Outdoor interventions should be considered in relation to each person’s current health condition, unique resources, needs, and wishes.
- Outdoor interventions should be developed and carried out in collaboration between the older adults themselves, their significant others, as well as all relevant professionals.
- All planned outdoor interventions should be documented in the older adults’ existing plans, such as care, rehabilitation, support and/or implementation plans, which are regularly followed up.

### 3.3 Policy area 3: The outdoor environment at residential care facilities for older adults

*Vision:* The outdoor environment is a natural part of the living environment and daily life in RCFs. All zones (see Figure 1) are represented and are sufficiently spacious to accommodate the variety of functions required by the older adults living there, their family and friends who visit, and the care workforce.

*Proposal a:* The National Board of Housing, Building, and Planning should issue general guidelines on open space and property layouts for RCF plots to support a range of activities. This is in addition to the Swedish Planning and Building Act (2010:900 Chapter 8, Section 9) (Swedish Parliament, 2010), which already states that there should be sufficiently large open spaces suitable for outdoor use on plots intended for housing. It is proposed that the RCF-specific general guidelines should address the following bullet points:

- Zones 1–4 should be represented and available for everyone living at RCF, in line with the clarification of the upcoming points.
- Zones 1–3: The interaction between the zones should be considered so that zones 2 and 3 are clearly visible and accessible from the indoor environment used daily by the older adults and the care workers.
- Zones 2–3: There should be recreational areas available in both zones 2 and 3 for all residents, in all departments of the RCF. These areas should be sized for the number of people who may be present there at the same time. The sizing should also ensure that older adults in need of assistive devices and/or personal support have sufficient space to move around smoothly and enjoy a comfortable stay in the area.
- Zones 1–4: It should be possible to move between and stay in all zones as comfortably, safely, securely, and independently as possible, throughout the year, regardless of the weather and regardless of the need for assistance and aids.
- Zones 1–4: The environment should offer a range of opportunities for experiences of nature and the possibility for care, support, and rehabilitation in zones 1–4.
- Zones 1–4: When establishing and renovating RCFs, opportunities for experiences of nature in all four zones should be considered from the start of the project (i.e., before procurement).
- Zone 4: Special consideration should be given to enabling interaction with other target groups in the community and the possibility for shared use of public environments in the surroundings.

*Proposal b:* The government takes the initiative to support the implementation of outdoor stay strategies across all RCFs in Sweden. The strategy should include the following principles regarding the maintenance and management of the outdoor environment:

- Zones 1–3: Each RCF should have a routine for managing and developing the outdoor environment as an arena for outdoor activities, including prevention, care, and rehabilitation interventions.
- Zones 1–3: The routine should be developed in collaboration between the property managers and the RCF operation.

### 3.4 Policy area 4: Knowledge transfer to care workers and managers in RCFs for older adults

*Vision*: All individuals working in RCFs, including those responsible for managing and maintaining the environments, as well as managers and decision-makers, possess the knowledge and skills needed to recognize and utilize the outdoor environment as a resource for health and wellbeing. A person-centered approach permeates the entire operation, and outdoor activities are an integrated part of daily life, the work environment, and the living environment.

*Proposal*:

- The government should take the initiative to support the transfer of knowledge about the importance of health-promoting outdoor environments, outdoor activities, and outdoor-based care work in the National Board of Health and Welfare’s Knowledge Guide aimed at care workers in care for older adults.
- The government should take the initiative to support the transfer of basic educational components concerning the importance of health-promoting outdoor environments, outdoor activities, and outdoor-based care work in all upper secondary education programs, vocational training, and education at universities and colleges that train care workers intended to work in areas such as care for older adults.

### 3.5 Ideas for implementation

The group work concerning the implementation of the strategy proposal resulted in four themes. The first theme, *Overall prerequisites for implementation*, is general and relates to the entire strategy proposal. The following three themes are more specific and are entitled: *Collaboration between municipalities and civil society*, *Knowledge dissemination*, and *Education*.

#### 3.5.1 Overall prerequisites for implementation

Each of the four policy areas must be addressed through political processes. Relevant parliamentary committees for each strategy area should review and decide on their respective parts of the proposal. In addition, certain national authorities must be empowered by the government to take on specific responsibilities, as follows:

- The National Board of Health and Welfare must ensure that older adults living in RCFs are offered time outdoors daily, both in the form of recreation and relaxation and in the form of care and rehabilitation. Preferences for outdoor engagement should be systematically documented in existing plans.
- The National Board of Housing, Building, and Planning must ensure the establishment of principles for usable open spaces related to the outdoor environment in RCFs for older adults.

Furthermore, the government must ensure that municipalities are given ownership responsibility for:

- Ensuring access to zones 1–4 according to the zone model.
- Political anchoring of key figures for usable open spaces in the outdoor environment.
- Including the outdoor environment in the local functional program (i.e., support in governance and specifying requirements).

#### 3.5.2 Collaboration between municipalities and civil society

Collaboration between the municipality and civil society (e.g., involving volunteers, family, and friends) needs to be strengthened to contribute to increased knowledge and volunteer activities. The municipalities should involve family organizations and encourage engagement and provide training for volunteers who wish and are able to facilitate outdoor activities in RCFs.

#### 3.5.3 Knowledge dissemination

There is a need for a freely accessible platform for the healthcare sector and the landscape sector where research and good examples from practice are presented, for example, via authorities’ websites. Furthermore, popular science reports are needed on preferred qualities in outdoor environments, on spending time outdoors, and on outdoor-based care and rehabilitation in RCFs. These reports should be aimed at decision-makers and politicians.

#### 3.5.4 Education

People engaged in the development of the present strategy proposal who are already working with secondary education, vocational education, and teaching at universities and colleges that train people who intend to work in areas such as care for older adults should raise the issue internally and with the Ministry of Education, and in their various professional associations. The professional associations should then, in turn, should raise the issue with politicians.

Furthermore, the government should assign the National Board of Health and Welfare and the National Board of Housing, Building, and Planning to develop training packages related to the national strategy. The training packages should be aimed at national, regional, and local decision-makers, politicians, and managers, as well as practitioners from both the healthcare and landscape sectors.

## 4 Discussion

In this study, we developed a proposal for a national strategy to support access to outdoor stays in residential care facilities for older adults, drawing on evidence from research on health-promoting outdoor environments and collaboration with a range of stakeholders. While the language used in the strategy may at times appear directive, its content should be understood as collectively generated through a co-creation process. The strategy has not yet been empirically tested or evaluated in practice and should therefore be regarded as a practice-informed proposal grounded in stakeholder dialogue and existing evidence.

This discussion opens by presenting a rationale for the strategy and then outlines the potential consequences of a lack of guidelines. The discussion primarily highlights the parts of this study that can be anchored in international research on person-centered care, on nature and health, as well as on strategy development work, and thus aims to demonstrate the generalizability of the results in an international context.

### 4.1 A national strategy—a path toward health equity and sustainability

Outdoor environments play a vital role in supporting the physical, psychological, and social wellbeing of older adults (de Keijzer et al., 2019; Xu et al., 2022). In line with this, an increasing number of countries around the world are working with outdoor activities to promote the health and wellbeing of older people (Chong et al., 2020; McNiel and Westphal, 2020; Piva et al., 2022). However, despite the growing evidence of benefits, outdoor access in RCFs is often limited, sporadic, and dependent on individual staff initiative or facility design. In the absence of national strategies, a policy vacuum has emerged around outdoor access in RCFs. This vacuum has resulted in inconsistent practices, where residents’ opportunities for outdoor engagement depend largely on individual care workers’ priorities, organizational factors, or physical infrastructure (Artmann et al., 2017; Liljegren et al., 2024a), rather than on a shared standard of care. The inconsistency in care provision can mean a systemic neglect of nature as a determinant for wellbeing and ultimately, can exacerbate health inequities among older people living in RCFs.

Although older adults wish to spend time outdoors (Liljegren et al., 2024b; Dahlkvist et al., 2016) and despite the fact that both residents and care workers in RCFs recognize the positive effect on health and wellbeing of spending time outdoors (Tavilsup et al., 2023; Liljegren et al., 2024a,2024b), many residents in need of assistance seldom get outside (Artmann et al., 2017). Reinforcing a care model focused on physical maintenance rather than holistic wellbeing means that the health-promoting potential of nature goes untapped. This can contribute to increased sedentary behavior (de Souto Barreto et al., 2016), perceived loneliness (Gardiner et al., 2020), and mental distress (Wang et al., 2024) among older adults in RCFs. Hence, national standards are crucial to prevent disparities among RCF residents and promote equitable and dignified aging, as well as high-quality of care for older adults. Moreover, the benefits of a national strategy extend beyond residents’ health. Care workers in RCFs often work under significant stress and time constraints (Josefsson et al., 2007; Costello et al., 2019; Väisänen et al., 2024). For them, structured outdoor work can provide restorative experiences that ease emotional strain and enhance the meaningfulness of care work, leading to improved staff wellbeing, reduced burnout, and increased job satisfaction. This, in turn, can lead to better staff retention (Evans et al., 2019), which is crucial to maintain a high quality of care in RCFs. Thus, a national strategy for access to outdoor environments and stays in RCFs is proposed as a way to promote conditions that may support both residents’ and care workers’ health, contributing to social, biological, and economic sustainability. Integrating outdoor access into RCFs reflects an approach to care that is inclusive, rights-based, and oriented toward both individual and collective health and wellbeing (World Health Organization [WHO], 2024).

### 4.2 Results with novel perspectives on person-centered care and contact with the outdoors

A strength of the strategy developed in this study is that its four policy areas comprehensively address the outdoor environment and outdoor activities as integrated aspects of everyday life in RCFs, and that it highlights the outdoor environment as a resource for the care and rehabilitation that the care workers provide. The significance of the outdoor environment and activities is multifaceted. On the one hand, it concerns health and wellbeing, while on the other hand, it concerns the possibility of person-centered care. Therefore, education for individuals who work or are being trained to work at or in connection with RCFs is a necessary part of the strategy.

This study clearly indicated that if care workers are to provide person-centered care, the outdoor environment must be an integral part of the care environment from the start. Therefore, to optimally promote opportunities for contact and relationships with the outdoor environment, the zone model of Bengtsson (2015) permeates the strategy throughout. The use of the zone model is particularly motivated by the target group of older adults in RCFs because they represent a group that particularly benefits from outdoor activities (Ottosson and Grahn, 2005, 2006; Dahlkvist et al., 2016; Sefcik et al., 2021), but also because they are very sensitive to the design of the environment (Lawton and Nahemow, 1973; Lien et al., 2015). Therefore, it is extremely important that the connection between indoors and outdoors is well thought out, so that older adults feel at home and understand how they can move between zones, making the entire environment a natural resource for them. When the outdoor environment in all zones is a natural part of the living environment and the care environment, truly person-centered care can be achieved. A well-considered interaction between the zones means, for example, that zones 2 and 3 are clearly visible from zone 1, i.e., in the environments where older adults spend most of their time indoors, which is confirmed by studies (e.g., Dahlkvist et al., 2016; van den Berg et al., 2020).

### 4.3 In collaboration toward a national strategy

Although this study focuses on older adults in residential care facilities (RCFs) and adopts a person-centered approach, older adults themselves were not directly included in the digital workshops, primarily due to practical and ethical considerations related to digital accessibility, consent procedures, and the organizational context of the participating facilities. However, their perspectives were systematically incorporated through the participation of care staff and family members, who contributed insights grounded in their close, day-to-day knowledge of older adults’ preferences, needs, and lived experiences. In addition, the strategy development was informed by existing empirical studies and background literature, including research on older adults’ experiences of care and outdoor environments.

This highlights the importance of co-creation as a collaborative process in which research-based knowledge, practice-based expertise, and indirectly represented perspectives of older adults are jointly integrated. In this study, such co-creation involved close collaboration between researchers and practitioners, such as care workers and those involved in the planning and development of RCF operations and the physical environments of RCFs, enabling research evidence and experiential knowledge to be translated into a detailed and practically relevant strategy proposal. The study did not include a formal assessment of sample adequacy, information power, or saturation, nor a systematic analysis of representativeness across stakeholder groups, which can be considered a limitation. However, the purposive sampling approach ensured the inclusion of stakeholders with relevant expertise and experience, contributing to a diverse and contextually grounded material. Furthermore, the iterative and participatory nature of the workshop process strengthened the study by enabling continuous dialogue, reflection, and the integration of multiple perspectives throughout the research process.

Therefore, although the number of participants decreased with the increasing demands of the work (see Table 1), in our opinion, the outcome resulted in a well-supported proposal with a satisfactory level of concretization for a national strategy. This was made possible, partly thanks to the approach of starting the workshop series, with the creation of common ground in such a way that participants at all levels, as determined by the multi-level model of Kislov et al. (2014), were given a voice. Furthermore, just as experienced in a research project by Allen et al. (2019), the iterative workshop process enabled articulation of a knowledge base and led to detailed and well-thought-out proposals and ideas. In addition, having two moderators working together in conducting the workshops, both with extensive experience of working with similar processes and contexts, was an advantage. To ensure equal participation and focus, they provided structured agendas with discussion prompts and prepared digital whiteboard templates. The digital format offered convenience by lowering thresholds for participation, such as traveling and scheduling constraints, and allowed individuals from different geographic locations and with different abilities to contribute. On the other hand, it may have hindered spontaneous dialog and the informal exchanges that often arise in physical meetings. To overcome technical barriers and problems with digital literacy, all participants were encouraged to e-mail additional thoughts and input in the intervals between the workshops.

Possibly, a setup with a shorter period for conducting the workshop series, which extended over one and a half years, would have been beneficial for maintaining engagement and reducing the number of participant dropouts in this study, which is in line with the guidelines for workshop processes described by Sufi et al. (2018). However, in this study, the working conditions of the researchers made a shorter period for conducting all three workshops impossible. Another potential drawback of the study lies in the composition of the participant group. Even though cross-sectoral representation was sought, there was a risk that certain voices were underrepresented. For instance, patient associations, as well as pensioners’ and carers’ organizations, were invited to participate, since their voices are particularly important to shed light on the lived experience. However, only very few individuals from these groups attended. Low digital competence may hinder the ability or willingness to engage with a digital format. On the other hand, many groups appreciate the inclusiveness that online workshops offer. One way to increase impact and democratic anchoring would be to have a formal consultation on the strategy proposal with these groups. Other voices that we sought to collect were those of the Public Health Agency of Sweden and the Swedish Association of Local Authorities and Regions. They were invited to take part in the workshop series but declined as there was no formal government mandate at the time. Their perspectives would have been valuable in lending additional weight and relevance to the proposal. Nevertheless, four other authorities (the National Board of Health and Welfare, the Swedish National Board of Housing, Building, and Planning, the Swedish Institute for Human Rights, and the Swedish Agency for Participation) participated in the study and provided valuable contributions, which is a strength of the proposal.

### 4.4 The importance of government support

As noted above, a mandate from the country’s government would have enabled and supported legitimacy, impact, and cross-sector coordination in the implementation of the strategy. A clear government mandate for strategy development ensures political anchoring, resources, and a structured implementation, which is ultimately crucial for the success of the strategy (Christensen and Lægreid, 2020; Nasiritousi and Grimm, 2022). Hence, it is a weakness that the strategy proposal was developed based on a research project and not at the request of the government. However, to obtain research funding today, a detailed plan for the implementation of the results is often required, and, in this case, it was such an incentive that led to the development of the strategy proposal. Nevertheless, political representatives from the Swedish Parliament have followed the workshop series with great interest and welcome the proposal as a basis for future decisions. Translating proposals into governmental or parliamentary decision-making typically involves extensive preparatory processes. The present study may offer a contribution to such a process by providing a co-created and evidence-informed strategy proposal grounded in stakeholder perspectives and existing knowledge. Thus, it is hoped that the national strategy proposed in this study may help inform and garner support from the Swedish Government for future policy development.

### 4.5 Practical applicability of the strategy proposal

In Sweden, implementation of the national strategy for outdoor access and outdoor stays could be linked to existing quality assurance and monitoring systems. At a national level, opportunities for outdoor access are already partly reflected in the National Board of Health and Welfare’s (Socialstyrelsen) annual user and RCF-unit surveys, which are answered by residents and unit managers. For instance, statistics from 2024 indicate considerable potential for improvement as 92% of the care providers lack routines for outdoor activities and 80% lack supportive conditions for movement to and within outdoor environments (Socialstyrelsen, 2024). By recognizing outdoor access and outdoor stays as a quality dimension of residential care, such existing indicators could provide a basis for systematic municipal follow-up and contribute to ongoing quality improvement efforts. Where relevant, indicators could also inform supervisory processes conducted by the Swedish Health and Social Care Inspectorate (IVO). In the absence of stronger national governance, responsibility for implementation will largely rest with local leadership in Sweden’s 290 municipalities, making municipal prioritization and organizational commitment key factors for successful implementation.

### 4.6 Study limitations

The study’s limitations have been discussed throughout the Discussion section but are summarized here to provide a coherent overview.

- The strategy proposal is informed by stakeholder dialogue and existing evidence and has not been empirically tested or evaluated in practice.
- While cross-sectoral representation was pursued, certain groups, including RCF residents and organizations representing pensioners and carers, were underrepresented in the workshops, and some institutional actors could not participate due to a lack of formal government mandate at the time.
- The absence of a clear government mandate represented a key limitation. As the strategy proposal was developed within a research project rather than initiated by the government, it lacked some of the political legitimacy, resources, and coordination needed for structured implementation, potentially limiting its future impact.

## 5 Conclusion

While this proposal was developed within the specific context of Sweden, its foundational principles and collaborative development approach offer potential applicability also in other national settings. The emphasis on cross-sectoral participation, digital co-creation, and person-centered values aligns with global trends in promoting healthy aging and combating ageism (World Health Organization [WHO], 2024). However, transferability requires careful consideration of contextual differences such as variations in healthcare systems, cultural norms, policies, and regulations. Even so, the proposal may serve as a conceptual point of departure for international adaptation and knowledge exchange, as the flexible and collaborative development approach allows for tailoring to diverse contexts. National adaptations should therefore involve local stakeholder engagement and be attentive to cultural and structural factors to support relevance and context-sensitive application.

## Data Availability

The raw data supporting the conclusions of this article will be made available by the authors, without undue reservation.
